# Risk factors of pathologic complete response for neoadjuvant chemoradiotherapy in locally advanced rectal cancer

**DOI:** 10.3389/fonc.2025.1483065

**Published:** 2025-07-18

**Authors:** Liangjing Zhou, Gaoyang Cao, Liming Shi, Chunrong Fei, Weifeng Lao

**Affiliations:** ^1^ Department of General Surgery, Sir Run Run Shaw Hospital, School of Medicine, Zhejiang University, Hangzhou, Zhejiang, China; ^2^ Department of Colorectal Surgery, Sir Run Run Shaw Hospital, School of Medicine, Zhejiang University, Hangzhou, Zhejiang, China; ^3^ Department of Radiation Oncology, Sir Run Run Shaw Hospital, School of Medicine, Zhejiang University, Hangzhou, Zhejiang, China; ^4^ Department of Clinical Laboratory, Sir Run Run Shaw Hospital, School of Medicine, Zhejiang University, Hangzhou, Zhejiang, China

**Keywords:** rectal cancer, pathologic complete response, neoadjuvant chemoradiotherapy, NLR, clinical factors

## Abstract

**Background:**

Global cancer statistics indicate colorectal cancer as the second leading cause of cancer-related deaths, with rectal cancer accounting for approximately 30% of cases. Despite neoadjuvant chemoradiotherapy (nCRT) being standard for locally advanced rectal cancer (LARC), only 10-30% of patients achieve pathologic complete response (pCR). The aim of this research was to identify variables for predicting pCR in rectal cancer patients after nCRT.

**Methods:**

This retrospective study analyzed 285 LARC patients treated with nCRT and total mesorectal excision (TME). Univariate and multivariate logistic regression was performed to identify the association between clinicopathological characteristics and pCR. A nomogram based on the univariate logistics regression was built to estimate the likelihood of pCR prior to treatment decisions.

**Results:**

Univariate logistic regression revealed a significant association between pCR and multiple factors, including histology, CEA levels, clinical N stage, circumferential resection margin (CRM), and the neutrophil-to-lymphocyte ratio (NLR). Upon further multivariate logistic regression, histology, CEA levels, and NLR emerged as the independent predictive factors. A predictive nomogram was developed based on these factors, achieving good predictive ability with an AUC of 0.786

**Conclusion:**

Clinical factors including histology, CEA levels, clinical N stage, circumferential resection margin, and NLR are important predictors of treatment response to nCRT for locally advanced rectal cancer. Furthermore, the developed nomogram aims to facilitate individualized and more effective treatment planning.

## Introduction

Global cancer statistics reveal colorectal cancer as the second most lethal malignancy worldwide (1). Rectal cancer comprises approximately 30% of all colorectal cancer cases, and this proportion has been steadily rising annually (2). However, the majority of rectal cancer patients are diagnosed at a locally advanced stage and have a poor prognosis (3). At present, the standard of treatment for locally advanced rectal cancer still recommends radical resection after neoadjuvant chemoradiotherapy. This approach aims to downstage the disease, enhance resection rates, and minimize local tumor recurrence. However, clinical observations highlight a sobering reality: among nCRT patients, only 10% to 30% achieve a pathologic complete response, while about 45% exhibit a partial response, and the remainder fails to show any notable improvement (4, 5).

However, for patients who are non-responsive to chemoradiotherapy, nCRT can unfortunately postpone the timing of radical surgery. Furthermore, some patients face the risk of overtreatment, which escalates treatment-related toxicities and undermines their immune function (6). Therefore, the accurate evaluation of the efficacy of nCRT in the treatment of rectal cancer is of great significance. TNM staging is considered to be the most effective evaluation of prognosis (7). However, TNM staging is not entirely accurate, as patients with identical tumor stages can exhibit varied clinical outcomes (8). In recent years, the oncology field has broadened its scope to encompass various novel tumor parameters for consideration, such as microsatellite instability (9), methylation (10), tumor-infiltrating lymphocyte (11), immune checkpoint (12), and the specific genes and signaling pathways (13). These biomarkers offer a more individualized understanding of tumor behavior, potentially enhancing treatment strategies and predicting outcomes.

The systemic inflammatory response (SIR) holds a significant correlation with the prognosis of various tumors (14). Neutrophils, lymphocytes, and albumin, serving as key markers of SIR, are closely related to tumor treatment. Walsh et al. uncovered that patients with elevated NLR exhibited an increased risk of recurrence and poorer prognosis in colorectal cancer (15). Both NLR and platelet lymphocyte ratio (PLR), being non-specific inflammatory markers, offering insights into the inflammatory response, as well as predicting the prognosis of tumors. Previous studies have shown that the elevated NLR and PLR levels before treatment of rectal cancer suggest poor prognosis (16). However, there are few studies exploring the potential of NLR and PLR in predicting pathological complete response after neoadjuvant chemoradiotherapy for rectal cancer.

Therefore, identifying clinical factors that correlate with achieving pCR following nCRT is of great importance. The aim of this research was to identify clinicopathological factors that serve as predictive markers for pCR in patients with locally advanced rectal cancer undergoing neoadjuvant chemoradiotherapy. Furthermore, we aimed to formulate a predictive nomogram that could accurately estimate the likelihood of achieving pCR prior to initiating treatment decisions, thereby facilitating individualized and more effective treatment planning.

## Materials and methods

### Patients

We conducted a retrospective analysis, spanning from 2010 to 2016, of 285 individuals diagnosed with locally advanced rectal cancer at the Sir Run Run Shaw Hospital of Zhejiang University College of Medicine. Inclusion criteria: (1) Patients with a pathologically confirmed diagnosis of rectal cancer; (2) clinical stage of T2-4, N0-2, M0; (3) distance to the anal verge ≤15 cm; (4) received nCRT combined with total mesorectal excision and postoperative adjuvant chemotherapy if necessary; (5) complete clinicopathological features, imaging, follow‐up, and clinical data. Patients with distant metastasis, as well as those harboring other malignant diseases were excluded from the study. This study was approved by the ethics committee of the Sir Run Run Shaw Hospital of Zhejiang University College of Medicine.

### Treatments

All patients enrolled in the study underwent nCRT with either intensity‐modulated radiotherapy (IMRT) or three‐dimensional conformal radiotherapy (3DCRT), complemented by oral administration of capecitabine during the radiotherapy period. The dose of radiotherapy was 30-50 Gy/25–28 times. Patients received neoadjuvant chemotherapy at the end of radiotherapy. Neoadjuvant chemotherapy protocol: mFolFox6 (calcium folinate 400 mg/m^2^, fluorouracil 2600 mg/m^2^, oxaliplatin 85 mg/m^2^) or Capeox (oxaliplatin 130 mg/m^2^, capecitabine 1000 mg/m^2^ bid). After completion of nCRT, for patients with T2 or T3 tumors without mesorectal fascia involvement, the TME was performed. For patients with T3 tumors involving the mesorectal fascia or T4 tumors, the TME combined with en bloc resection of adjacent tissues or organs was conducted to ensure an adequate surgical margin. Additionally, for patients with T3-T4 or N1-N2 tumors located below the peritoneal reflection, the extended lateral pelvic lymph node dissection was incorporated into the TME (17, 18). The resected specimens underwent histopathological examination immediately following the surgery. The depth of tumor infiltration and lymph node metastases were assessed according to the AJCC TNM staging system, with histological diagnosis serving as the gold standard.

### Statistical analyses, model development, and validation

The primary endpoint for developing the dynamic nomogram was pCR. Differences between cohorts were analyzed using chi‐squared and *t*‐tests. Logistic regression analysis was employed to identify independent predictors for pCR. *p* < 0.05 was considered statistically significant. The nomogram was constructed based on the findings from the univariate logistic regression analysis. The evaluation of the model was based on a consistency index (C‐index) to determine accuracy, a decision curve analysis (DCA) to assess clinical benefit, and a calibration curve to assess the consistency of the model’s predicted results with the actual results. The Delong test was used to compare the area under the curve (AUC). SPSS version 26.0 and R 4.1.1software were used for statistical analysis.

## Results

### Patients’ clinicopathological characteristics

**Table 1** presents the clinicopathological characteristics of the patients. This study involved a total of 285 patients, with a median age of 59.4 years. The cohort included 193 males and 92 females. Using the TNM staging system, the counts for T2, T3, and T4 stages were 28, 243, and 14, respectively, whereas the corresponding numbers for N0, N1, and N2 stages were at 70, 132, and 83. The most common histological subtype was adenocarcinoma, comprising 88.07% of cases. In terms of biomarkers, 54.23% (n=109) of patients tested positive for CEA, while 12.94% (n=26) were found to be CA199-positive. At the initial staging, 27.37% of all tumors exhibited a positive circumferential resection margin. The tumor locations were distributed as follows: 8.77% in the lower rectum, 51.58% in the middle rectum, and 39.65% in the upper rectum. A total of 90.53% (n=258) of patients received higher radiation doses, whereas 9.47% (n=27) received lower doses. Additionally, 60.70% (n=173) of patients underwent radiochemotherapy in conjunction with sodium glycididazole treatment, while 39.30% (n=112) did not. A latency period spanning over 8 weeks was observed in 15.44% (n=44) of patients from completion of radiotherapy to surgery. Conversely, 82.11% (n=234) of patients exhibited a latency period ranging from 6 to 8 weeks, while a minority of 2.46% (n=7) patients experienced a latency period of less than 6 weeks. A total of 52.63% (n=150) of patients were categorized into the high NLR group, whereas 47.37% (n=135) of patients were stratified into the low NLR group.

**Table 1:** Patients and treatment characteristics.

Variables	Total (n=285)	Non_pCR (n=223)	pCR (n=62)	*P*
Age, n(%)				0.759
≤ 60	152 (53.33)	120 (53.81)	32 (51.61)	
> 60	133 (46.67)	103 (46.19)	30 (48.39)	
Gender, n(%)				0.542
Male	193 (67.72)	153 (68.61)	40 (64.52)	
Female	92 (32.28)	70 (31.39)	22 (35.48)	
Histology, n(%)				0.017
Adenocarcinoma	251 (88.07)	191 (85.65)	60 (96.77)	
Mucinous adenocarcinoma	34 (11.93)	32 (14.35)	2 (3.23)	
CEA, n(%)				<.001
≤ 5.0	92 (45.77)	52 (36.88)	40 (66.67)	
> 5.0	109 (54.23)	89 (63.12)	20 (33.33)	
CA199, n(%)				0.205
≤ 37.0	175 (87.06)	120 (85.11)	55 (91.67)	
> 37.0	26 (12.94)	21 (14.89)	5 (8.33)	
T stage, n(%)				0.125
2	28 (9.82)	18 (8.07)	10 (16.13)	
3	243 (85.26)	195 (87.44)	48 (77.42)	
4	14 (4.91)	10 (4.48)	4 (6.45)	
N stage, n(%)				0.003
0	70 (24.56)	45 (20.18)	25 (40.32)	
1	132 (46.32)	106 (47.53)	26 (41.94)	
2	83 (29.12)	72 (32.29)	11 (17.74)	
CRM, n(%)				0.010
Negative	207 (72.63)	154 (69.06)	53 (85.48)	
Positive	78 (27.37)	69 (30.94)	9 (14.52)	
NLR, n(%)				<.001
≤ 3.5	135 (47.37)	87 (39.01)	48 (77.42)	
> 3.5	150 (52.63)	136 (60.99)	14 (22.58)	
Tumor location, n(%)				0.955
Lower rectum	25 (8.77)	19 (8.52)	6 (9.68)	
Mid rectum	147 (51.58)	115 (51.57)	32 (51.61)	
Upper rectum	113 (39.65)	89 (39.91)	24 (38.71)	
Radiation dose, n(%)				0.159
≤ 46Gy	27 (9.47)	24 (10.76)	3 (4.84)	
> 46Gy	258 (90.53)	199 (89.24)	59 (95.16)	
Sodium glycididazole, n(%)				0.487
No	173 (60.70)	133 (59.64)	40 (64.52)	
Yes	112 (39.30)	90 (40.36)	22 (35.48)	
Latency, n(%)				0.252
< 6	7 (2.46)	7 (3.14)	0 (0.00)	
6-8	234 (82.11)	184 (82.51)	50 (80.65)	
> 8	44 (15.44)	32 (14.35)	12 (19.35)	

**Figure 1:**
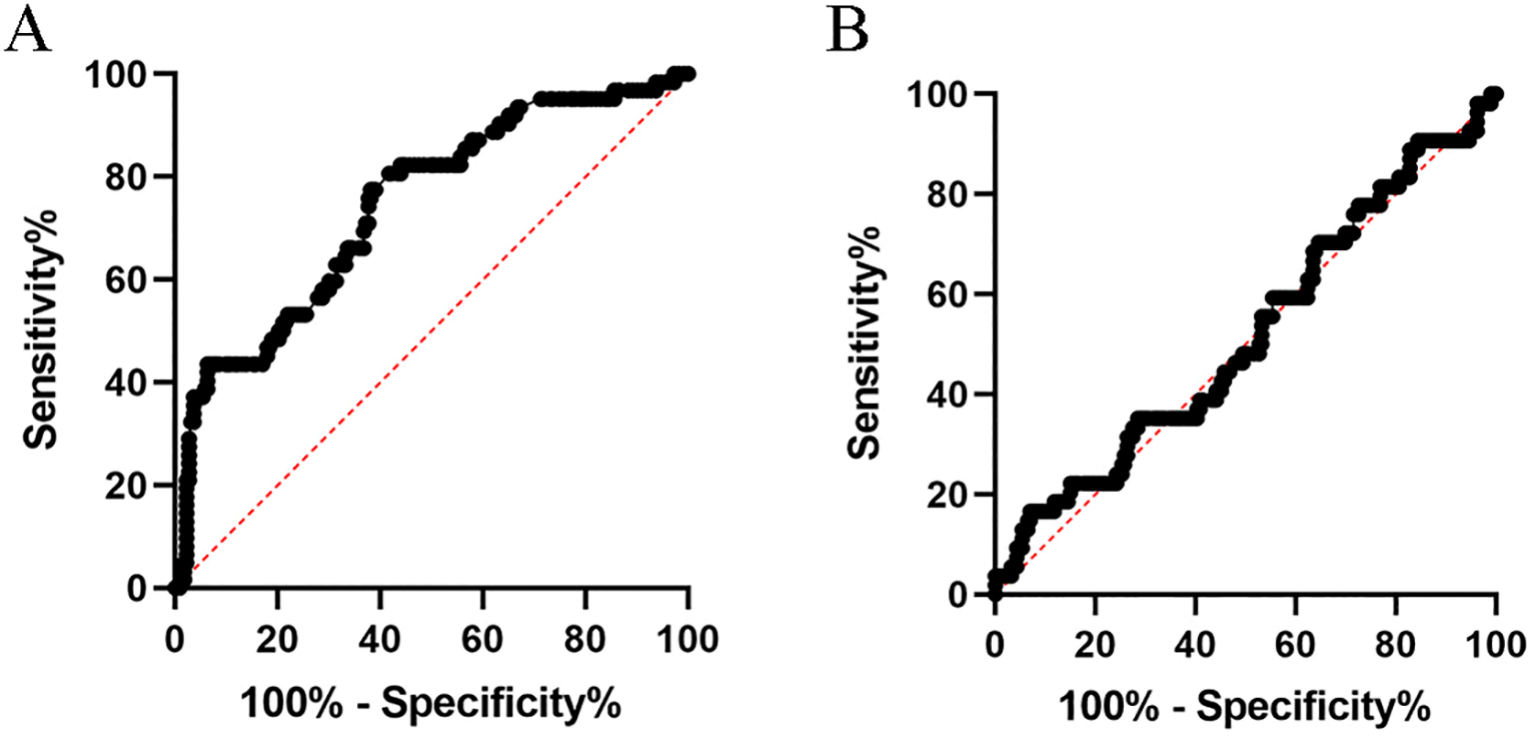
ROC Curve showing the predictive value of NLR **(A)** and PLR **(B)** for pathologic response to nCRT in locally advanced rectal cancer.

The association between pCR and clinicopathological characteristics was also presented in **Table 1**. The proportion of patients with pCR was 21.8%, with 223 non-pCR and 62 pCR cases. The χ^2^ test or Fisher’s exact test was used for comparisons. The optimal cut-off values for NLR and PLR were identified through the receiver operating characteristic curve analysis, subsequently stratifying patients into high and low groups based on these values. **Figure 1A** represents the ROC curve for NLR, exhibiting an AUC of 0.742 and an optimal cutoff point of 3.5. Conversely, **Figure 1B** depicts the ROC curve for PLR, revealing an AUC of 0.516. Among the variables, pCR was significantly associated with histology (p<0.05), CEA levels (p<0.001), clinical N stage (p<0.05), circumferential resection margin (p<0.05), and NLR (p<0.001). However, no significant correlation was observed with age, gender, CA199 levels, clinical T stage, tumor location, radiation dose, use of sodium glycididazole, or the latency period between the pCR and non-pCR groups.

### Univariate and multivariate logistic regression analyses of pCR

According to the univariate logistic regression analysis showed in **Table 2**, patients diagnosed with common adenocarcinoma were significantly more likely to achieving pCR compared to those with mucinous adenocarcinoma (OR = 0.2; 95% CI: 0.05–0.85; P = 0.030). Patients with higher clinical N stages were significantly less likely to reach pCR (OR = 0.44; 95% CI: 0.23–0.85; P = 0.014 for N1 and OR = 0.28; 95% CI: 0.12–0.61; P = 0.002 for N2, respectively). Additionally, patients with positive CEA levels had notably lower odds of achieving pCR (OR = 0.29; 95% CI: 0.15–0.55; P < 0.001). The presence of a positive circumferential resection margin also significantly decreased the likelihood of pCR (OR = 0.38; 95% CI: 0.18–0.81; P = 0.013). Intriguingly, patients with higher NLR values were more likely to attain pCR (OR = 0.19; 95% CI: 0.10–0.36; P < 0.001), suggesting a potential association between inflammation and treatment response.

In the multivariate analysis in **Table 2**, the independent predictive factors that remained significant were histology (OR = 0.20; 95% CI: 0.04–0.99; P = 0.048), CEA levels (OR = 0.41; 95% CI: 0.20–0.84; P = 0.015), and NLR (OR = 0.24; 95% CI: 0.11–0.50; P < 0.001), each maintaining their respective predictive roles.

**Table 2:** Univariate analyses and multivariate analyses of predictors for pCR using logistic regression models.

Variables	Univariate analyses		Multivariate analyses	
	OR (95%CI)	*P*	OR (95%CI)	*P*
Age				
≤ 60	1.00	0.759	1.00	0.753
> 60	1.09 (0.62 ~ 1.92)		1.12 (0.56 ~ 2.25)	
Gender				
Male	1.00	0.542	1.00	0.726
Female	1.20 (0.66 ~ 2.17)		0.88 (0.42 ~ 1.83)	
Histology				
Adenocarcinoma	1.00	0.030	1.00	0.048
Mucinous adenocarcinoma	0.20 (0.05 ~ 0.85)		0.20 (0.04 ~ 0.99)	
CEA				
≤ 5.0	1.00	<.001	1.00	0.015
> 5.0	0.29 (0.15 ~ 0.55)		0.41 (0.20 ~ 0.84)	
T stage				
2	1.00		1.00	
3	0.44 (0.19 ~ 1.02)	0.056	0.79 (0.26 ~ 2.38)	0.672
4	0.72 (0.18 ~ 2.90)	0.644	1.37 (0.22 ~ 8.45)	0.731
N stage				
0	1.00		1.00	
1	0.44 (0.23 ~ 0.85)	0.014	0.55 (0.24 ~ 1.22)	0.142
2	0.28 (0.12 ~ 0.61)	0.002	0.46 (0.16 ~ 1.32)	0.147
CRM				
Negative	1.00	0.013	1.00	0.528
Positive	0.38 (0.18 ~ 0.81)		0.72 (0.26 ~ 1.99)	
NLR				
≤ 3.5	1.00	<.001	1.00	<.001
> 3.5	0.19 (0.10 ~ 0.36)		0.24 (0.11 ~ 0.50)	

### Establishment and validation of nomogram model

**Figure 2:**
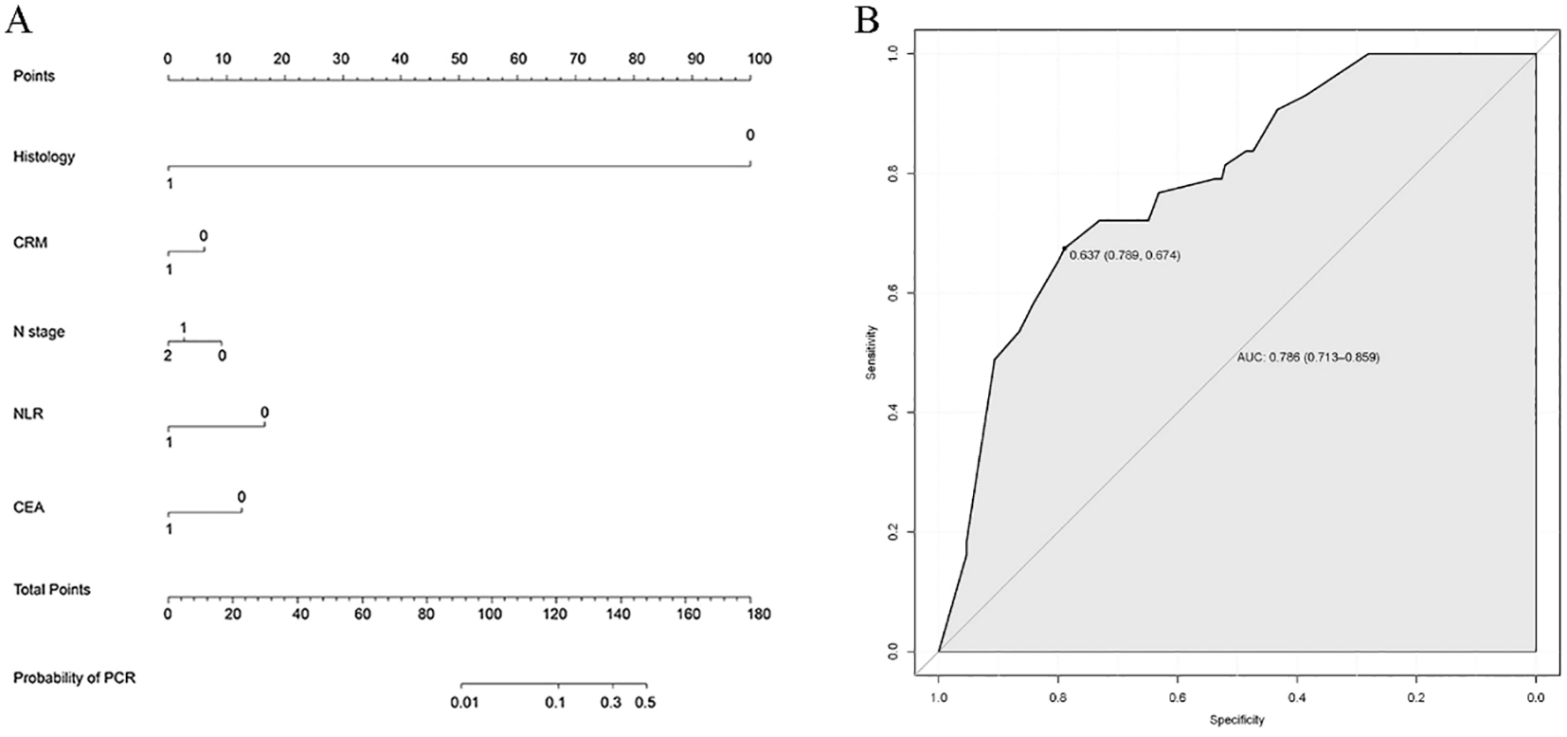
Establishment and validation of nomogram model. **(A)** Nomogram to predict the probability of achieving pCR. The factors of histology, CEA levels, clinical N stage, CRM status, and NLR were included in the model. **(B)** ROC curve of the nomogram in the training cohort.

To predict the occurrence of pCR subsequent to neoadjuvant chemoradiotherapy for rectal cancer, we incorporated factors exhibiting statistically significant differences in the univariate logistic regression analysis, namely, histology, CEA levels, clinical N stage, CRM status, and NLR, into the development of a nomogram (**Figure 2A**). Each category within each factor was assigned a score, and the total score obtained by summing the scores of each factor corresponds to the probability of achieving pCR. The AUC value of the ROC curve is 0.786 (95% CI 0.713–0.859), indicating good predictive ability (**Figure 2B**).

## Discussion

Rectal cancer is a common cancer worldwide. However, approximately half of all patients are diagnosed with locally advanced cancer, which has a higher risk of recurrence and mortality (19). The implementation of neoadjuvant radiochemotherapy, in conjunction with total rectal mesorectal excision as the standard treatment, has significantly enhanced the prognosis in the management of rectal cancer (20). Our findings reveal that among LARC patients undergoing nCRT, the rate of pCR was 21.8%, which was similar to previous research (21). Among the variables, pCR was significantly correlated with histology, CEA levels, clinical N stage, circumferential resection margin, and NLR. However, factors such as age, gender, CA199 levels, clinical T stage, tumor location, radiation dose, use of sodium glycididazole, or the latency period failed to predict pCR. These findings provide valuable insights for identifying individuals who are more likely to benefit from preoperative therapy, thereby facilitating personalized treatment strategies.

Cancer-related local and systemic inflammation has emerged as a pivotal factor in tumor invasion and metastasis. Numerous studies have highlighted the significance of various biomarkers and hematological indices, including NLR and PLR, as indicative markers of the local immune response. Lee et al. demonstrated that NLR and PLR correlate with pCR or primary tumor downstaging subsequent to nCRT in colorectal cancer patients (22). However, our current study revealed that just NLR was associated with pCR post nCRT for rectal cancer. Notably, NLR can be acquired through routine blood tests, yet there remains a lack of consensus regarding an optimal cutoff value. Kim et al. chose 3 as the cutoff based on the predictive ability in terms of pathological tumor response and survival analysis (23). In this study, we chose 3.5 as the cutoff according to the Youden index. Consequently, determining the most appropriate cutoff value is crucial for precisely forecasting pathological tumor response and patient prognosis following nCRT.

Serum carcinoembryonic antigen (CEA) was widely used as a biomarker, playing a pivotal role in diagnosing cancer and assessing the efficacy of treatment. The diagnostic and prognostic value of this marker has undergone rigorous evaluation in numerous studies (24). Recent researches have explored its potential as a predictive indicator of tumor response to neoadjuvant therapy in rectal cancer patients (25). Wallin et al. conducted a study involving 530 patients and discovered, through multivariate analysis, that low CEA levels were significant predictors of pathologic complete response (26). Meanwhile, Yang et al. demonstrated that low-level CEA before nCRT was predictive factors for pCR of preoperative neoadjuvant therapy for locally advanced rectal cancer (27). Inspired by these findings, our research endeavor analyzed the relationship between serum CEA and response to nCRT. Our multivariate logistic regression analysis also confirmed that CEA level emerged as a predictive factor for achieving pCR on the treatment response to nCRT in patients with LARC.

The clinical N stage is an established prognostic factor in rectal cancer, influencing both the likelihood of achieving a pCR and overall survival outcomes. Patients with higher N stages are less likely to achieve pCR, as evidenced in our study and supported by previous literature (28, 29). Higher N stages reflect a more advanced disease, with increased lymph node involvement suggesting a more aggressive tumor biology and a reduced response to neoadjuvant therapy. This finding underscores the importance of precise nodal staging in the pre-treatment evaluation of rectal cancer patients.

The status of the circumferential resection margin is another critical predictor of pCR and long-term outcomes in rectal cancer. CRM involvement is defined as tumor touching or within 1 mm from the outermost part of tumor or outer border of the mesorectal or lymph node deposits to the resection margin. Among the radiological evaluations, MRI stands out due to its high accuracy in evaluating T and N staging and specificity in detecting CRM involvement (30). A positive CRM is associated with a higher risk of local recurrence and poorer survival rates (31), making it a significant factor in surgical planning and post-operative treatment strategies. Our study confirms that a positive CRM significantly decreases the likelihood of achieving pCR. This is consistent with existing evidence that emphasizes the need for achieving clear resection margins to improve oncologic outcomes. Achieving a negative CRM during surgery is crucial for minimizing the risk of residual disease and recurrence (32, 33).

Mucinous adenocarcinoma of the rectum was reported to be a poor indicator for nCRT in terms of larger residual tumors, higher incidence of margin positivity, and greater residual nodal disease (34). Our study also included patients to examine the prevalence of a low pCR rate among individuals diagnosed with mucinous adenocarcinoma, and this finding remained consistent in the regression analyses. Consequently, the application of the watch-and-wait strategy in mucinous adenocarcinoma patients should be cautiously employed, given the low pCR rates observed.

The efficacy and safety of different radiotherapy doses in neoadjuvant chemoradiotherapy for LARC has been extensively explored. This study employed conventional radiotherapy doses (30-50 Gy/25–28 times), with no significant correlation observed between treatment response and radiation dose. Consistent with these findings, Xu et al. demonstrated that within conventional dose ranges, higher radiotherapy doses did not confer a better survival outcome. In the 50.4Gy group, the rates of pCR and cCR were 19.4% and 12.5%, respectively, while in the 45Gy group, the rates were 22.9% and 10.4%, showing no statistical significance. Notably, while patients receiving 50.4 Gy demonstrated a higher rate of anal retention, this regimen was associated with increased incidence of adverse events including radioactive proctitis, myelosuppression, and intestinal obstruction or perforation (35). Recent studies have brought increasing attention to the potential role of radiation dose intensification in LARC (36). Elisa Bertocchi et al. conducted a comparative analysis between standard dose radiotherapy (SDR, 50.4 Gy/28 times) and radiation dose intensification (RDI, 60 Gy/30 times), and the primary endpoint of the study was the pCR rate. Although the RDI group showed a significantly higher primary tumor downstaging in T3 tumors, no statistically significant difference was observed in pCR rates (20.8% vs 22.6%). Notably, acute genitourinary toxicity was significantly higher in RDI group (37). In conclusion, current evidence suggests that radiation dose intensification in preoperative chemoradiotherapy for LARC appears feasible. Therefore, strategies to enhance radiation therapy response through dose optimization should be further pursued.

Limitations of this study include patient selection bias and unavailable variables that may potentially be related to pCR. Firstly, given its retrospective cohort analysis, there exists a degree of selection bias, potentially leading to either an underestimation or overestimation of the probability of pCR when patients with incomplete clinical data are excluded. Second, when selecting patients at the beginning of our study, we may exclude those with incomplete data or who had not completed nCRT. In addition, recent studies have demonstrated that the watch-and-wait (W&W) strategy may be safe for LARC patients with a clinical complete response (cCR), through endoscopic or radiological assessment. This approach potentially obviates the morbidity associated with surgery (38). However, cCR does not always correlate with pCR, posing challenges in accurately selecting patients for the W&W strategy. As the research on colorectal cancer continues to deepen, novel prognosis-related variables, including BRAF, RAS gene status, and microsatellite stability, will gradually be identified. Besides, Yothers et al. developed the neoadjuvant rectal cancer score (NAR) using yielded pathological N (ypN) and downstaging of T (clinical T – pathological T) based on relative weights suggested by the nomograms, which was validated as a predictor of overall survival following neoadjuvant therapy for rectal cancer (39). Consequently, the nomogram model utilized for predicting pCR should be regularly updated to incorporate more new factors and ensure a more precise and accurate prediction assessment.

Taken together, clinical factors including histology, CEA levels, clinical N stage, circumferential resection margin, and NLR are important predictors of treatment response to nCRT for locally advanced rectal cancer. Furthermore, we have devised a predictive model to assess the likelihood of pCR prior to making treatment decisions. With these clinical indicators, we aim to facilitate the development of individual treatment strategies for patients with LARC.

## Data Availability

The original contributions presented in the study are included in the article/supplementary material. Further inquiries can be directed to the corresponding authors.
